# On-Ground Testing of Dual-Sided Release Mechanism of TianQin Test Mass Using a Pendulum

**DOI:** 10.3390/s25092878

**Published:** 2025-05-02

**Authors:** Ji Wang, Diwen Shi, Chao Xue, Biao Yang, Bingwei Cai, Jie Chang, Zefan Zhou, Wenhai Tan, Shanqing Yang

**Affiliations:** MOE Key Laboratory of TianQin Mission, TianQin Research Center for Gravitational Physics & School of Physics and Astronomy, Frontiers Science Center for TianQin, Gravitational Wave Research Center of CNSA, Sun Yat-sen University (Zhuhai Campus), Zhuhai 519082, China; wangj689@mail2.sysu.edu.cn (J.W.); shidw5@mail2.sysu.edu.cn (D.S.); yangb99@mail2.sysu.edu.cn (B.Y.); caibw3@mail2.sysu.edu.cn (B.C.); changj9@mail2.sysu.edu.cn (J.C.); f14zhou@163.com (Z.Z.); tanwh7@mail.sysu.edu.cn (W.T.); yshq@mail.sysu.edu.cn (S.Y.)

**Keywords:** TianQin project, the locking-and-release mechanism, on-ground testing, transferred momentum, pendulum system

## Abstract

The high-precision gravitational reference sensor, which hosts a heavy test mass (TM) surrounded by electrodes with a relatively large gap, is crucial in all high-sensitivity drag-free sensors. Consequently, a dedicated locking mechanism is needed to securely hold the TM during the launch phase. After reaching the intended orbit, the TM is released to a free-falling state and subsequently captured by electrostatic actuation, which demands that the transferred momentum and angular momentum to the TM do not exceed $10^{-5}\;\mathrm{kgm}/s$ and $10^{-7}\;{\mathrm{kgm}}^{2}/s$, respectively. This paper introduces a three-level structural design of the locking-and-release mechanism. In order to investigate the release requirement, a pendulum system has been developed for on-ground testing. The mock-up of the TM is entirely consistent with the size and mass of TianQin TM, and the dual-sided release tips constrain the TM and then rapidly retract simultaneously, after which the transferred momentum and angular momentum are estimated from the free oscillations as $0.38\left(21\right)\times 10^{-5}\;\mathrm{kgm}/s$ and $0.15\left(14\right)\times 10^{-7}\;{\mathrm{kgm}}^{2}/s$ with a preload force of 0.3 N. This proposes a feasible scheme for validating the release mechanism conducting impulse testing for the TianQin project.

## 1. Introduction

In numerous scientific space missions, particularly those that involve gravitational phenomena, a massive free-falling object is frequently utilized as a standard reference for detecting. This object is employed in inertial sensors, which are instruments that measure and record physical quantities such as velocity, acceleration, and position, such as DISCOS [1], GP-B [2], CHAMP [3], GRACE [4], GOCE [5], Microscope [6,7], LISA [8,9,10,11], TianQin [12,13,14], and so on. The TianQin project aims to detect gravitational waves in the frequency range $10^{-4}$–1 Hz through a space mission. Establishing a high-precision gravitational reference sensor (GRS), which serves as the free-falling test masses (TMs) for laser ranging, is a crucial technological objective.

To meet the scientific objectives of detecting gravitational waves (GWs) from the reference source RX J0806.3+1527 (J0806) [15], from which the GW strain is estimated to be $h_{0}∼6.4\times 10^{-23}$, the non-gravitational disturbance on the TM needs to be reduced to the order of $10^{-15}$ m/s^2^/${\mathrm{Hz}}^{1/2}$ in millihertz frequencies [12]. To achieve a signal-to-noise ratio (SNR) of 10 within 3 months of observation, we expect for the laser interferometer to achieve a peak positional sensitivity $\sqrt{S_{x}}\approx 1$ pm/${\mathrm{Hz}}^{1/2}$ at 6 mHz. The requirement on the residual acceleration $\sqrt{S_{a}}$ must satisfy the most stringent acceleration sensitivity requirement to ensure it does not mask the J0806’s GW signal (6 mHz), which is shown as follows:

(1) $$\frac{h_{0}\sqrt{T}}{10}\geq \frac{2}{\sqrt{R_{0}}}{\left[\frac{S_{x}}{L_{0}^{2}}+\frac{S_{a}}{{\left(2\pi f\right)}^{4}L_{0}^{2}}\left(1+\frac{10^{-4}\mathrm{Hz}}{f}\right)\right]}^{1/2},$$

where $R_{0}$ is the transfer function at a frequency of 6 mHz, $L_{0}∼10^{-5}$ km is the arm length, *T* represents a continuous observation duration of 90 days and *f* is 6 mHz. The TianQin project plans to adopt a gold–platinum alloy cubic TM with a mass of approximately 2.45 kg and a volume of 50 × 50 × 50 mm^3^. The gaps between the TM and the electrode frame are designed to be 4–5 mm.

During the launch phase, the entire payload is subjected to large accelerations and adequate protection must be provided to prevent damage to the TM and electrode frame. After releasing, the TM is captured by the electrostatic control system to ensure the safety and reliable operation of the GRS and the normal functioning of the gravitational wave detector. Hence, a specialized locking-and-release mechanism is imperative for securely holding the TM during transit and subsequently releasing it prior to the scientific phase. Furthermore, the large gap between the TM and the electrode frame limits the electrostatic control capability, with a driving force and torque of approximately $5\times 10^{-7}$ N and $1\times 10^{-8}$ Nm, respectively. Due to the limited electrostatic force, strict limitations must be imposed on the released TM state, requiring that the transferred momentum of TM be less than $10^{-5}$ kgm/s and the angular momentum be less than $10^{-7}$ kgm^2^/s. Such a requirement is to guarantee that the limited electrostatic control force/torque can control and stabilize the TM in the center of the electrode frame, avoiding impacts with the surroundings.

To verify the release phase on the ground, the University of Trento in Italy has designed and developed a Transfer Momentum Measurement Facility (TMMF) to measure the momentum transferred to the TM during the release process under representative conditions of mass release to free fall in the LISA Pathfinder test. An approximately 100 g plate-shaped TM model was suspended as a simple pendulum, and a release tip model and a blocking system were employed to contact and release the TM model [16,17,18,19,20]. In Huazhong University of Science and Technology, a measurement system based on a compound pendulum has been designed and built, which uses two beryllium copper plates to suspend a lightweight aluminum TM model. The experiment used stainless steel and aluminum tips for locking and releasing from opposite positions, respectively, and the release momentum was obtained from the occupation of the pendulum [21]. Our team has performed a three-level structural design of the locking-and-release mechanism for TianQin. In order to test the releasing requirement, a pendulum system for on-ground testing has been developed. The mock-up of the tungsten TM is entirely consistent with the size and mass of the TianQin TM, with dual-sided release tips constraining the TM and then quickly retracting. Furthermore, the designed retractable release mechanism is positioned on both sides of the TM mock-up. The contact surface material is also identical to the contact surface material in the orbital environment. By analyzing the transferred momentum to the TM mock-up with dual-sided tips, the contribution of the release actions of the two tips during the orbital phase to the residual velocity of the TM can be predicted. This kind of pendulum system, which is closer to the flight configuration, allows us to measure both the simple pendulum and torsion balance modes of the suspended test mass and calculate the released momentum and angular momentum. These experiment results provide valuable guidance for the design of the TianQin TM locking-and-release mechanism.

## 2. The Locking-and-Release Mechanism of TianQin

### 2.1. The Purpose of Locking and Releasing

**Figure 1:**
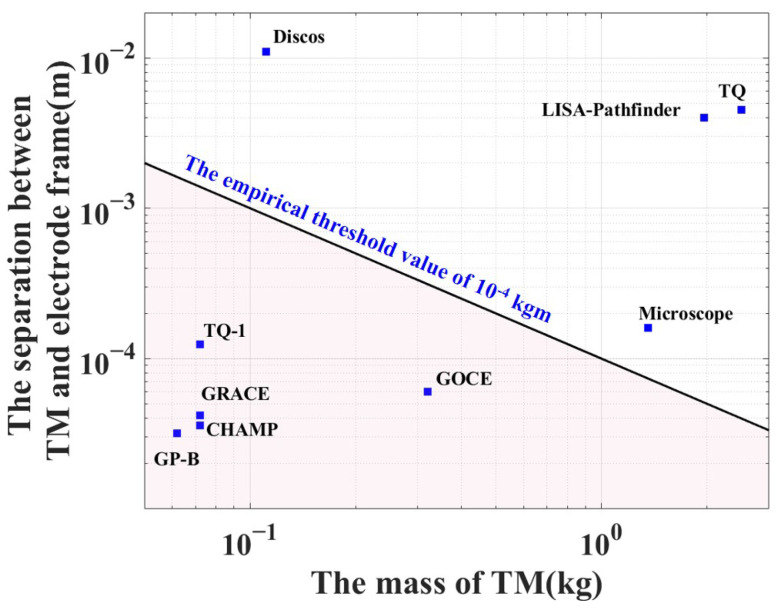
The empirical threshold value which depends on the mass and separation for different space missions [1,2,3,4,5,7,11,12,13]. The space missions above the blue line requires the usage of the locking-and-release mechanism for protecting the TM.

Due to the massive mass of the TM and its large separation from the electrode frame, the unconstrained TM will have significantly higher impact kinetic energy $K_{impact}$ during the launch phase [22], shown as

(2) $$K_{impact}=\frac{1}{2}mv_{impact}^{2}=\frac{1}{2}m\left(2ad\right)=amd,$$

where *m* is the mass of the TM; $v_{impact}$ is the impact velocity; *a* is the launcher acceleration, which is approximately the same exhibited in typical space missions; and *d* is the potential free motion distance. The impact kinetic energy primarily depends on the product of the mass and the separation between the TM and the electrode frame, as shown in Figure 1. Consequently, an empirical threshold value of $10^{-4}$ kgm has been established to understand whether a locking system is needed in inertial sensors. If the value falls below the threshold, the impact energy remains within an acceptable range, making it possible to avoid adopting a locking system. Exceeding the empirical threshold will lead to damage from impacting that can affect the space missions. Therefore, the caging-and-vent mechanism (CVM) is designed to constrain the TMs during launch phase and later transitioned to the grabbing, positioning and release mechanism (GPRM), which is used to release the TMs to the free-falling state [23,24,25,26]. For example, the inertial sensor of TQ-1 satellite [13] carries a 70 g cubic Ti-alloy TM with a capacitive separation of about 145 μm along the high-sensitivity axes, and there is no need to equip the locking-and-release mechanism. The core instrument of the LISA-Pathfinder consists of two Au-Pt alloy cubic TMs with a mass of about 2 kg and gaps from electrostatic shield of 2.9–4.0 mm on the different axes [10], where a locking mechanism is required. In the case of the TianQin project, the product with a mass of 2.45 kg and gap of about 5 mm greatly surpasses the critical threshold, suggesting potential damage to the TM and electrode frame during the impact process. To ensure the secure and dependable transition of the inertial sensors into the scientific operational mode and guarantee the proper functioning of the TianQin space-borne gravitational wave detector, a dedicated locking-and-release mechanism for the TM is necessary, taking into account the launch conditions and the requirements.

### 2.2. The Main Structure of the Locking-and-Release Mechanism

**Figure 2:**
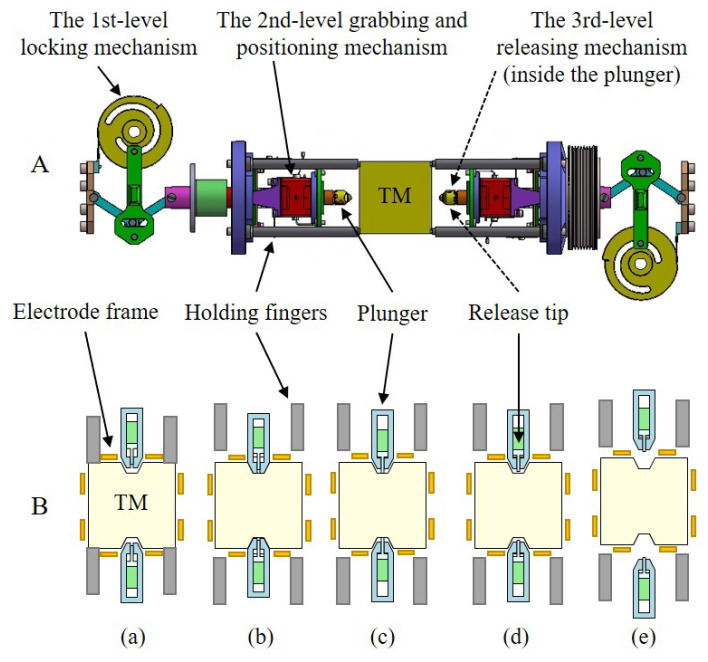
The main structure of prototype design for the locking-and-release mechanism of the TianQin project (**A**). A schematic diagram of the release process (**B**). (**a**) Locking of the TM by holding fingers (grey). (**b**) Positioning the TM with plungers (light blue) to the center of the electrode frame. (**c**) Handing over the TM to the release tips. (**d**) Releasing the TM by quickly retracting the RTs (green). (**e**) The released TM is captured and controlled by the electrostatic system (orange).

During the launch phase, the entire payload experiences significant random acceleration reaching up to 20 times the acceleration of gravity as 20 g. By applying the 3σ criterion, a minimum force of 1500N≈2.5kg×3×20 g is necessary for the unilateral locking of the TM. A prototype design for the locking-and-release mechanism of the TianQin project, depicted in Figure 2A, has been performed. This design adopts a symmetrical three-level structure, aimed at achieving gradual release and minimizing disturbances throughout the release process [23]. The first-level locking mechanism holds the TM tightly with eight holding fingers throughout the launch phase until reaching the designated orbit, and then the TM is handed over to the second-level grabbing-and-positioning mechanism with the restraining force of approximately 5 N for precise positioning. Subsequently, the two plungers of the grabbing and positioning mechanism retract, reducing the contact force to around 0.3 N, and gradually transferring the TM to the third-level releasing mechanism equivalently, which consists of a small release tip (RT) that is located inside and coaxial with the plunger. In order to achieve a smooth transition from the plungers to the tips and break the mechanical contact between the plungers and the TM, the maximum contact load of the RT on the TM is approximately 0.3–0.5 N. Finally, the TM is rapidly released with “zero” initial velocity through the retraction of RTs and captured and controlled by an electrostatic driving system. If the released state of the TM satisfies the electrostatic control criteria, the plunger retracts behind the electrode frame. The principle of release process (a)–(e) is shown in Figure 2B.

The staged release process offers the advantage of gradually reducing the preload force and contact area, which minimizes the effects of the final stage release action. When the TM and the release mechanism on both sides come into contact, the asymmetry of the release tip action may still significantly affect the release effect. In order to ensure the smooth progress of the task, it is necessary to establish stringent requirements for the post-release state of the TM and perform on-ground verification tests to ensure the stable release status.

### 2.3. The Requirements for the TM Release

**Figure 3:**
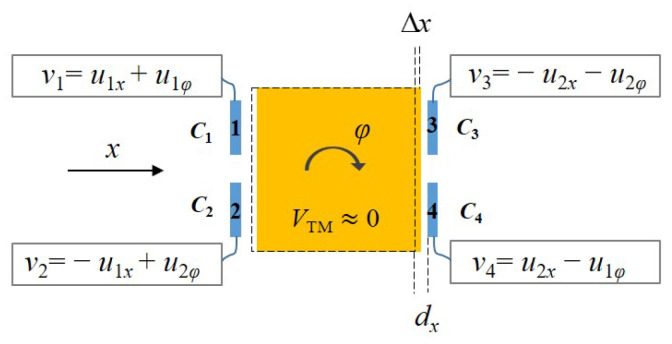
The capacitance between the TM and the electrode frame in the “*x*” direction, and the applied voltages.

The gravitational inertial sensor primarily comprises a massive TM and an electrode frame, which consists of 18 capacitor electrodes positioned approximately 4–5 mm away from the surfaces of TM on the different axes. The 12 electrodes are dedicated to the drive and control, while the remaining 6 electrodes are allocated for voltage biasing and sensing. As the TM moves a distance Δx in the “*x*” direction, as shown in Figure 3, the capacitance between the TM and the electrode frame is

(3) $$C_{0}=\varepsilon_{0}\frac{S}{d_{x}},$$

(4) $$C_{1}=\varepsilon_{0}\frac{S}{d_{x}+x}=C_{0}\frac{1}{1+\frac{x}{d_{x}}},$$

(5) $$C_{4}=\varepsilon_{0}\frac{S}{d_{x}-x}=C_{0}\frac{1}{1-\frac{x}{d_{x}}},$$

where $\varepsilon_{0}$ is the vacuum permittivity, *S* is the single-electrode area of about 400 ${\mathrm{mm}}^{2}$, and $d_{x}$ is the distance between the two surfaces of approximately 4 mm. The inductive bridge is connected between electrodes $C_{1}$ and $C_{4}$, and the difference between the two capacitors is calculated as

(6) $$\Delta C=C_{1}-C_{4}=2C_{0}\frac{\Delta x}{d_{x}}{\left[1-{\left(\frac{\Delta x}{d_{x}}\right)}^{2}\right]}^{-1}.$$

Gravitational inertial sensors can be categorized into two modes based on their sensing and control capabilities: high resolution (HR) and wide range (WR). HR mode is dedicated to scientific tasks, with a small driving force and sensing range, as well as extremely low sensing noise. The WR mode focuses on post-release sensing and TM electrostatic capturing tasks, providing several millimeters of sensing range. It has greater driving force and sensing range, at the cost of relatively high sensing noise. High-precision analog-to-digital conversion (ADC) collects the electrode voltage as a scientific voltage output, with a full-scale range of ±2.5 V and a corresponding WR mode equivalent capacitance range of ±2.5 pF, and all axes have the same capacitance range requirement [27]. The displacement range requirement in the most stringent direction is then established at approximately ±2 mm in WR mode.

In order to account for the decrease in distance between the electrode frame and the TM resulting from both TM translation and rotation, the displacement measurement range requirement of WR mode is distributed across the translational and rotational degrees of freedom uniformly. Meanwhile, an engineering safety factor of 2 has been selected. As a result, the displacement range requirement for the released TM includes two components: a maximum translation range of ±500 μm in three directions and an equivalent maximum rotational motion range of ±5 mrad.

In Figure 3, the application of driving voltages to electrodes 1, 2, 3, and 4 induces electrostatic forces on the TM. By applying AC voltages with varying frequencies to these electrodes, the coupling effects of electrostatic forces generated by different degrees of freedom can be mitigated or eliminated. Consequently, this enables independent control over the translational and rotational degrees of freedom of the TM. Further details regarding the optimal frequency of the AC signal employed for this purpose are extensively discussed in reference [28]. The applied voltages on the four electrodes are represented as $v_{1}$, $v_{2}$, $v_{3}$, and $v_{4}$, respectively, with $u_{1x}$ and $u_{2x}$ controlling the translation motion along the *x*-axis, and $u_{1\varphi }$ and $u_{2\varphi }$ controlling the rotational motion around the *z*-axis. Different-frequency AC signals are used for $u_{x}$ and $u_{\varphi }$, and there should be a definite phase difference between the two AC voltages.

According to the instantaneous electrostatic equation, the formulas for the electrostatic force and torque [29] generated by the AC voltages applied to the four electrodes on the TM are

(7) $$F=\frac{1}{2}\sum_{j=1}^{4}\frac{\partial C_{j}}{\partial q}{\left(V_{TM}-V_{j}\right)}^{2},$$

(8) $$M=\frac{1}{2}\sum_{j=1}^{4}\frac{\partial C_{j}}{\partial \varphi }{\left(V_{TM}-V_{j}\right)}^{2},$$

where $C_{j}$ is the capacitance between the TM and the electrode frame, $V_{j}$ represents the AC voltage applied to the plate, and $V_{TM}\approx 0$ V.

By differentiating the capacitance with respect to *x* and φ and ignoring small first-order terms, the expressions for the electrostatic force and torque can be obtained as

(9) $$F_{x}=\frac{1}{2}\frac{\varepsilon S}{d_{x}^{2}}\left(u_{1x}^{2}+u_{1x}^{2}-u_{2x}^{2}-u_{2x}^{2}\right),$$

(10) $$M_{\varphi }=\frac{1}{2}\frac{\varepsilon SR}{d_{x}^{2}}\left(u_{1\varphi }^{2}+u_{1\varphi }^{2}-u_{2\varphi }^{2}-u_{2\varphi }^{2}\right),$$

where ε is the dielectric constant of the medium between the two surfaces. The maximum electrostatic force and torque can be obtained when $u_{1x}$ and $u_{1\varphi }$ are at their maximum peak voltages and $u_{2x}$ and $u_{2\varphi }$ are at 0 V. The peak driving voltage of the electrodes is 140 V [28,30]. However, the sinusoidal voltages that generate the force and torque are not applied to the electrodes simultaneously at the maximum voltage. Instead, when using the electrostatic force to capture the TM, the sinusoidal signals for force and torque are alternately applied in every 100 ms, resulting in a duty cycle of 0.5 and half of the electrostatic force and torque on the TM. Moreover, there are also capacitance gradients between other conductors in the electrostatic system of the inertial sensor. The actual electrostatic force and torque are reduced by approximately 30% [27]. Therefore, the electrostatic forces in each degree of freedom can be calculated to be approximately $1\times 10^{-6}$ N, and the electrostatic torques in each degree of freedom are approximately $1\times 10^{-8}$ Nm.

When the TM is released, it typically possesses linear velocity and angular velocity. Additionally, there exist initial release positional and angular deviations relative to the center of the electrode frame. According to the effect of electrostatic forces and torques, the linear velocity and angular velocity must be diminished to near-zero within the allowed maximum motion range. The most stringent direction requirement is used as the criterion for release to ensure capturing safety and maximizing uniformity of requirements for each axis. Considering both the release position and velocity requirements, the typical target for release-transferred momentum is $10^{-5}\;\mathrm{kgm}/s$, while the typical parameter for release-transferred angular momentum is $10^{-7}\;{\mathrm{kgm}}^{2}/s$. Table 1 lists the TM release state requirements for each condition to ensure that the electrostatic control system can capture the TM.

## 3. The On-Ground Testing of the TM Released on Both Sides

### 3.1. Basic Principle of On-Ground Testing

**Figure 4:**
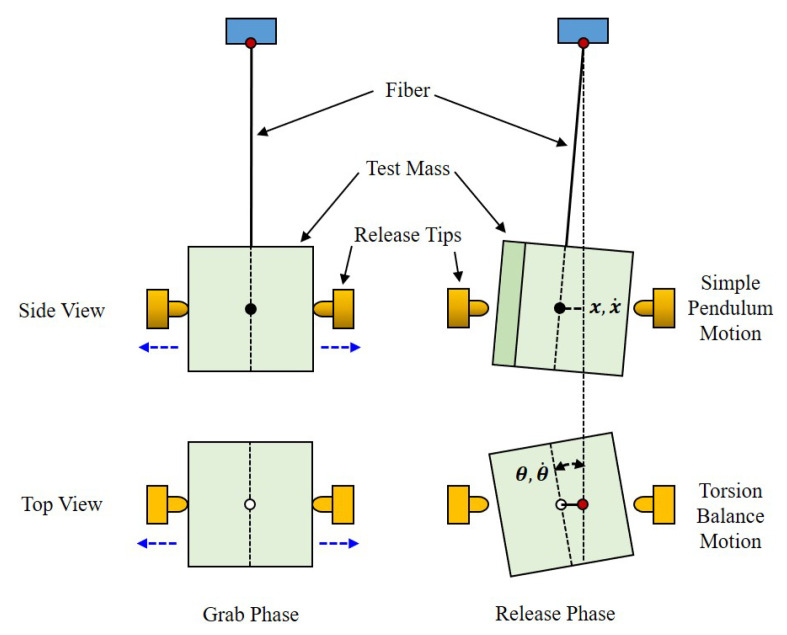
Basic principle of on-ground testing.

The measurement principle of the on-ground testing of the TM released on both sides is shown in Figure 4. The mock-up of the TM with the mass *M* and moment of inertia *J* is suspended by a thin fiber with length *L* as a pendulum system. Two release tips grab the TM on both sides and then retract simultaneously. The TM acquires the velocity $\dot{x}$ and angular velocity $\dot{\theta }$. Therefore, the transferred momentum and angular momentum from the RT to TM can be calculated as

(11) $$I_{x}=M\dot{x},$$

and

(12) $$L_{\theta }=J\dot{\theta }.$$

For the pendulum system in the small amplitude regime, the dynamic equation of motion of the pendulum can be expressed as

(13) $$M\ddot{x}+\gamma \dot{x}+kx=0,$$

where *x* is the displacement of the simple pendulum motion, γ is the damping factor, and *k* is the stiffness of the simple pendulum, respectively. The natural frequency of the simple pendulum is $\omega_{0}=\sqrt{\frac{k}{M}}$, and Equation (13) can be expressed as

(14) $$\ddot{x}+2\beta \dot{x}+\omega_{0}^{2}x=0,$$

where $\beta =\frac{\gamma }{2M}$ is the damping coefficient. The solution of the simple pendulum motion is shown as

(15) $$x=x_{m}e^{-\beta t}\cos\left(\omega_{x}t+\varphi \right),$$

where $x_{m}$ is the initial amplitude of the pendulum motion, $\omega_{x}=\sqrt{\omega_{0}^{2}-\beta^{2}}$ is the damping oscillation frequency, and φ is the initial phase. After releasing with the two tips, it is considered that TM acquires the initial displacement $x_{0}$ and initial velocity ${\dot{x}}_{0}$ at t=0 moment.

Based on Equation (15), $\dot{x}$ can be expressed as

(16) $$\dot{x}=x_{m}\left[-\beta \frac{x_{0}}{x_{m}}+\omega_{x}\sqrt{1-{\left(\frac{x_{0}}{x_{m}}\right)}^{2}}\right],$$

where the damping coefficient β can be expressed with the quality factor of simple pendulum $Q_{x}$ as $\frac{\omega_{x}}{2Q_{x}}$. Therefore, Equation (16) can be substituted into Equation (11) and obtain

(17) $$I_{x}=Mx_{m}\left[-\beta \frac{x_{0}}{x_{m}}+\omega_{x}\sqrt{1-{\left(\frac{x_{0}}{x_{m}}\right)}^{2}}\right].$$

Similarly, the angular velocity is

(18) $$\dot{\theta }=\theta_{m}\left[-\zeta \frac{\theta_{0}}{\theta_{m}}+\omega_{\theta }\sqrt{1-{\left(\frac{\theta_{0}}{\theta_{m}}\right)}^{2}}\right],$$

and the transferred angular momentum of the torsion balance system to the TM can be expressed as

(19) $$L_{\theta }=J\theta_{m}\left[-\zeta \frac{\theta_{0}}{\theta_{m}}+\omega_{\theta }\sqrt{1-{\left(\frac{\theta_{0}}{\theta_{m}}\right)}^{2}}\right],$$

where $\theta_{0}$, $\theta_{m}$, ζ, and $\omega_{\theta }$ are the initial angle, initial amplitude, damping coefficient, and damping oscillation frequency of the torsion balance motion, respectively. By measuring the periodic motion of the TM after release, the initial momentum transferred to the TM at the moment of release can be accurately calculated, which determines whether subsequent tasks can proceed smoothly.

### 3.2. Structure of the Release Mechanism

**Figure 5:**
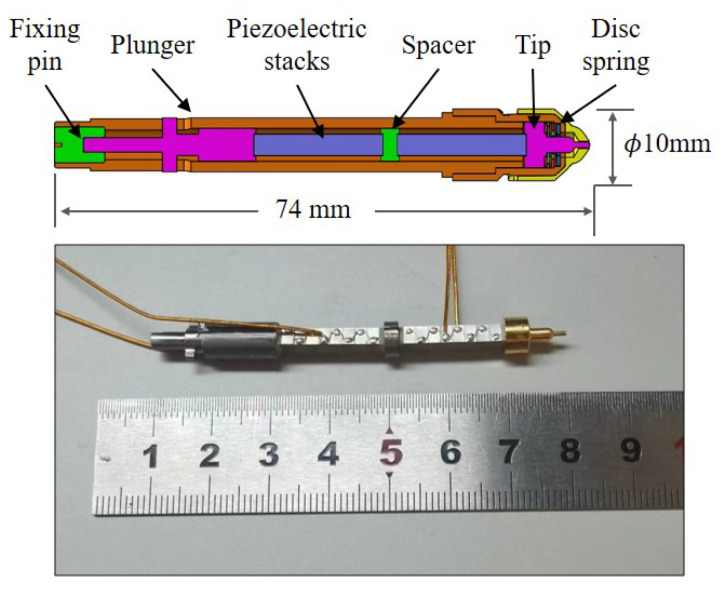
Structure design of the release mechanism and a photo of the piezoelectric tip.

The release mechanism, which will be adopted in locking-and-release mechanism of the TianQin project, has been designed and assembled for use in on-ground testing as shown in Figure 5. The small spherical-edged tip with a curvature radius of 1 mm and a diameter of 0.8 mm is constructed from titanium alloy with a gold-coated surface, coaxial with the grabbing hollow plunger, and compacted by a set of disc springs. During the release process, the tip is swiftly driven by a piezoelectric stack actuator, providing a maximum displacement of approximately 18 μm. In order to ensure the successful execution of the release procedure, another identical actuator serving as a backup is connected to the former with a spacer. A specially designed fixing pin is used to prevent the tip from rotating during linear retract movement. When a voltage of about 60 V is applied to the piezoelectric stack, the tip will move forward to the TM surface. While the power supply is turned off, the tip retracts rapidly with a maximum velocity of about 40 mm/s.

Any asymmetry in the retraction motion of the two opposite tips will cause residual forces to be exerted on the TM, leading to the transfer of momentum and angular momentum, which are caused by misalignment between the two tips in conjunction with a time delay between release. Misalignment in both horizontal and vertical directions can cause TM rotation. Therefore, from the deflection angle requirements in Table 1, it can be calculated that the release symmetry requirement is a tip alignment deviation of less than 200 μm. When there is a time delay between releases, the residual load of the tip on the TM will be converted into a push, and its contribution to the momentum is calculated as follows:

(20) $$I_{push}=F_{push}\Delta \tau ,$$

where $F_{push}$ is the residual load and Δτ is the time interval. The expected worst-case residual load is about 10 mN. To achieve the requirement of transferred momentum, the time interval between the retraction of the two tips is calculated to be less than 1 ms.

### 3.3. Design of Measurement

**Figure 6:**
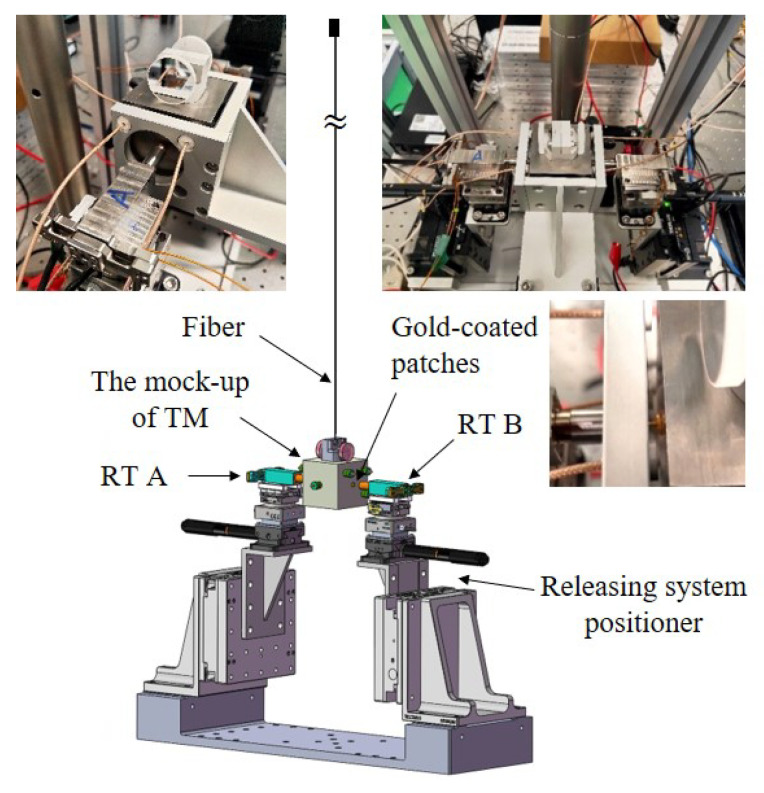
A schematic diagram of the on-ground testing platform. The mock-up of TM with gold-coated patches is suspended as a pendulum and released through dual-sided tips with gold-coated spherical-edged surfaces, which are adjusted in their position by the releasing system positioner. Eddy current displacement sensors and autocollimator are used to measure the translational and rotational signals of the TM.

To evaluate the performance of the releasing mechanism and investigate the impact of the dual-sided release tips, an on-ground testing platform has been designed and constructed, as shown in Figure 6. The testing experiment employs a mock-up TM, consistent with the size and mass of the TianQin TM. It also uses the TianQin dual-sided release tips, which enhances the consistency and representativeness of the testing. The main subsystems of the test activity are highlighted as follows:

- The suspended mock-up of TM: A tungsten cubic block with a length of 49.926(1) mm, width of 50.100(1) mm, and height of 50.089(2) mm is used as the TM, which is suspended by a tungsten fiber measuring 200 μm in diameter and 920 mm long. In order to ensure that the contact conditions between the surfaces of the TM and RT remain consistent with the on-orbit, the gold-coated patches are equipped on both sides of the TM. The total mass of the mock-up of the TM is 2375.005(1) g. The theoretical periods of oscillation of the simple pendulum motion and the torsion balance motion are 1.928(5) s and 38.5(2) s, respectively. Furthermore, different masses of the mock-up of the TM have also been employed to verify the release mechanism.
- The release mechanism: The structure of the release mechanism is designed and shown in Figure 5. Two RTs are actuated using a piezoelectric stack to make contact with the TM mock-up from both sides. Subsequently, the RT is quickly retracted to release the suspended TM.
- Releasing system positioner: The alignment of the release mechanisms on both sides, as well as the alignment between the release mechanisms and the TM, is accomplished using displacement stages and rotary stages with a resolution of 0.01 μm and 50 arcsec, respectively.
- Measurement system: The multiple eddy current displacement sensors are distributed on both sides of the TM to jointly measure the translational and rotational signals of the TM. Each sensor achieves a measurement resolution of 0.1 μm. An autocollimator is utilized to measure the pitch and yaw signals of the TM, providing a measurement accuracy of ±0.2 arcsec. The measurement resolution and the accuracy mentioned above are determined based on the fundamental principles of ground testing, reaching a momentum of $10^{-6}\;\mathrm{kgm}/s$ and an angular momentum of $10^{-8}\;{\mathrm{kgm}}^{2}/s$, which ensures measurement redundancy.
- Testing environment: The on-ground testing platform is placed on an optical isolation table to effectively isolate the influence of ground vibrations. The entire setup is enclosed in an acrylic enclosure to mitigate the effects of gas flow. A thermometer with an accuracy of 0.01 °C is utilized to concurrently monitor temperature variations throughout the experimental procedure.

To thoroughly characterize the release performance of the mechanism and ensure consistency in release outcomes, multiple experimental sets have been conducted, measuring the TM under varying contact preload conditions.

### 3.4. Experimental Preparation

**Figure 7:**
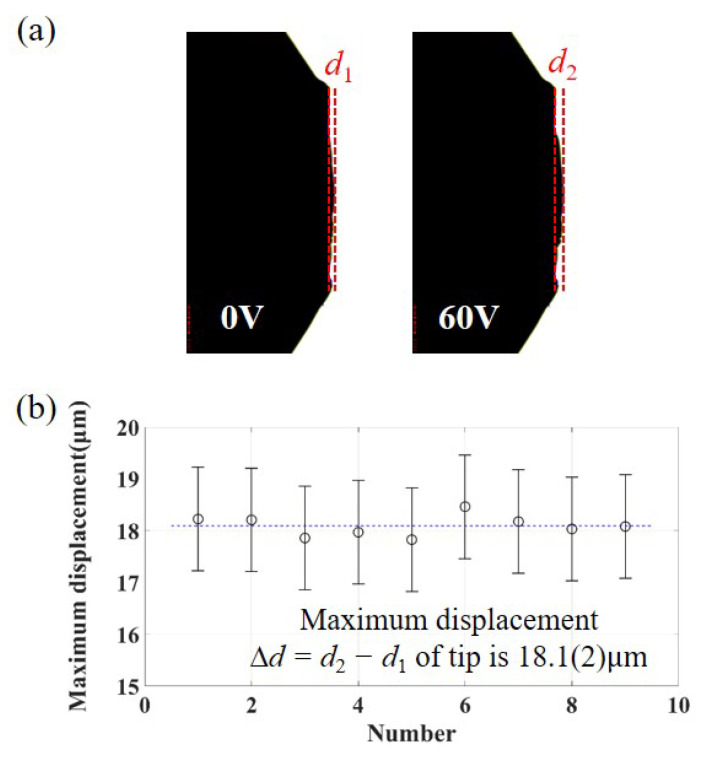
The maximum displacement of the tip. (**a**) Image of tip motion under the image-measuring instrument when the input voltage is 0 V($d_{1}$) / 60 V($d_{2}$); (**b**) the data of the maximum displacement $\Delta d=d_{2}-d_{1}$ of the tip.

The retraction of the RT is controlled by the input voltage applied to the piezoelectric stack. Before the formal experiment, the RT motion tests are conducted on the release mechanism. The assembled piezoelectric release mechanism relies on an external preload provided by a disc spring. When the input voltage 60 V reaches the maximum rated value of the piezoelectric stack, the reproducibility of the tip displacement is measured by an image-measuring instrument, illustrated in Figure 7. The maximum displacement of the tip is 18.1(2) μm.

**Figure 8:**
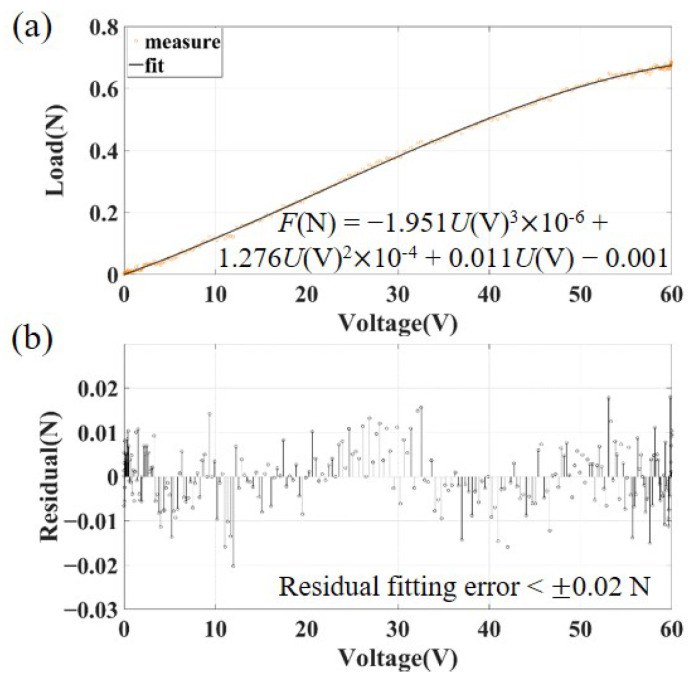
Measurement of preload force applied by the tip: (**a**) relationship between applied voltage and output force of release mechanism with the least square fitting; (**b**) the residual fitting error of the applied voltage and output force.

The preload force of the release tip that is applied to the TM is calibrated with the force sensor beforehand. The relationship between the voltage applied to the release mechanism and the resulting output preload force is shown in Figure 8a, and fitted with the least square method to obtain the expression, $F\left(N\right)=-1.951U{\left(V\right)}^{3}\times 10^{-6}+1.276U{\left(V\right)}^{2}\times 10^{-4}+0.011U\left(V\right)-0.001$, with a residual fitting error of less than ±0.02 N, as shown in Figure 8b. The high-order term in the fitting mainly refers to the hysteresis of piezoelectric continuous motion, and the fitting residual mainly refers to the uncertainty of the load sensor.

**Figure 9:**
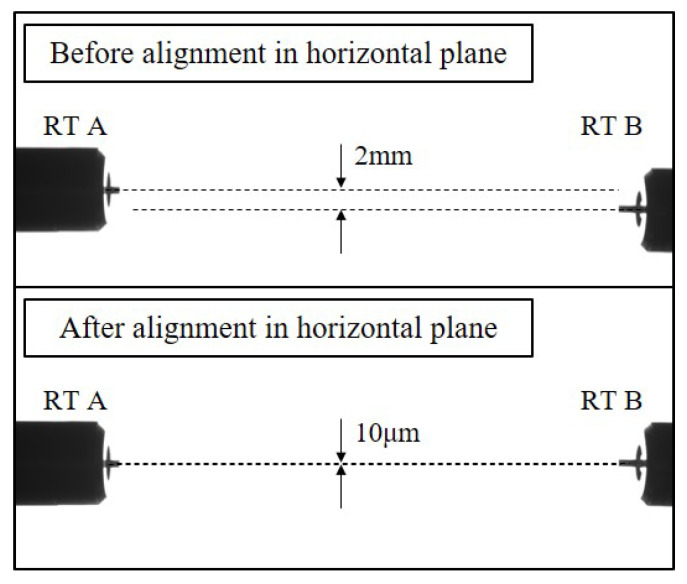
The typical images of the two tips in horizontal plane before and after alignment.

In the on-ground testing, the precision alignment of the dual-sided tips can be adjusted by the releasing system positioner, and measured with the industrial cameras with an accuracy of 10 μm. One camera is horizontally positioned to capture the image of the tips in the vertical plane, while another one is vertically positioned to capture the image in the horizontal plane. For each directional measurement, a standard reference gauge block is employed to calibrate the pixel size at a known distance, facilitating the calculation of the relative spatial positions based on the number and location of pixels occupied by the tips in the captured images. The images of dual-sided tips in horizontal plane before and after alignment are shown in Figure 9. The final alignment results indicate that the gap in the horizontal plane of the centerline of each release tip is 10 μm, and in the vertical plane is 18 μm. The well-aligned dual-sided release mechanism, along with the base, is assembled with the suspended TM mock-up to ensure the preservation of the relative spatial positions of RTs throughout the whole release process. The TM mock-up is adjusted and precisely positioned with the center of the dual-sided tips.

**Figure 10:**
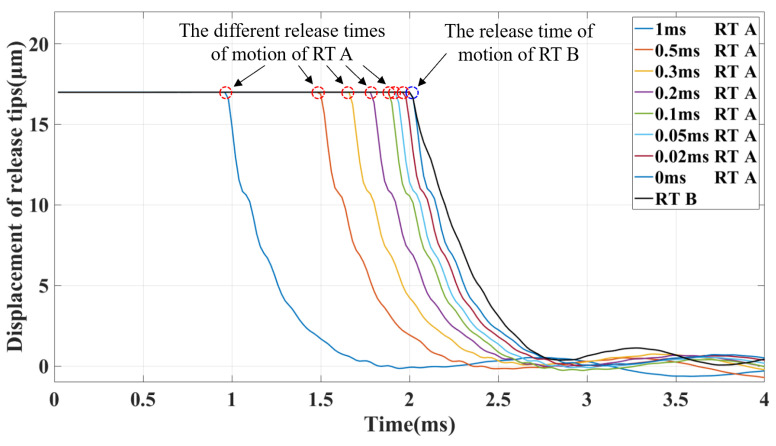
Test for asynchronous retraction of two tips within a time interval of 1 ms.

The response of the tips under different time intervals was verified using laser interferometry. The motion of the tips on both sides after the power was turned off was simultaneously measured. The time resolution was 0.02 ms, as the sampling rate of the laser measurement was 50 kHz. The displacement response of the tips under different time interval commands is shown in Figure 10. Accurate measurement of the time interval ensured the accuracy and reliability of the asymmetric release experiment. It was also helpful to estimate the potential influence of residual force.

**Figure 11:**
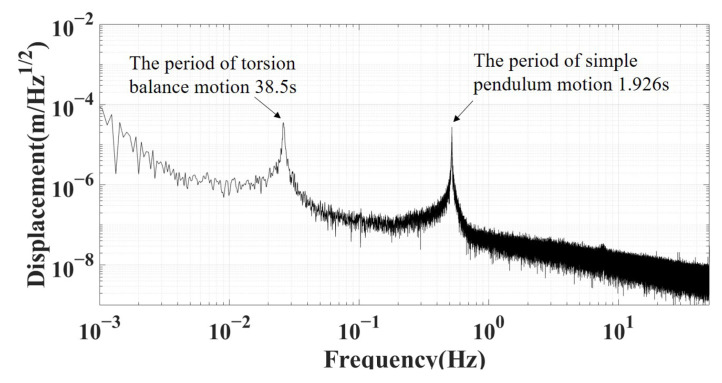
The power spectral density of the free oscillation of displacement signal.

The eddy current sensor has then been used to measure the TM free oscillation over a long time scale. The power spectral density of the displacement signal is shown in Figure 11. The measured period of simple pendulum and torsion balance are found to be 1.926 s and 38.5 s, respectively, which are consistent with the theoretical values within the error range.

## 4. Results

**Figure 12:**
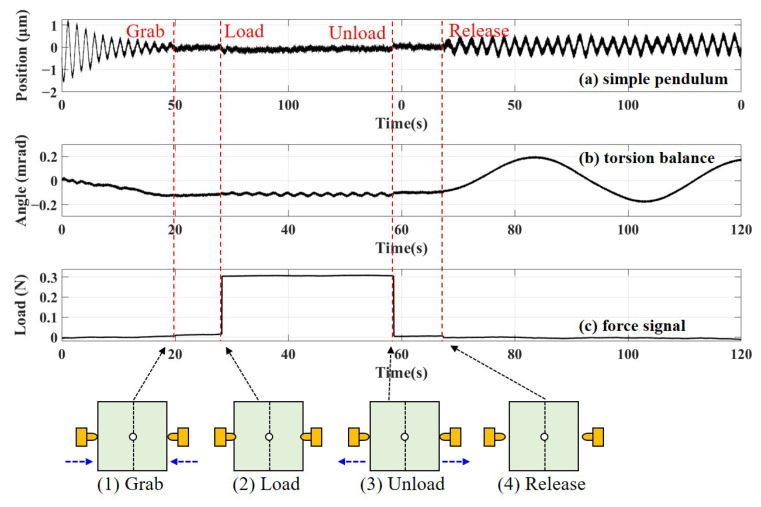
The typical data of (**a**) simple pendulum motion, (**b**) torsion balance motion, and (**c**) force signal diagram. Additionally, the four main stages of the release procedure of the TM: (**1**) Two tips grab the TM. (**2**) The preload force of 0.3 N is applied on the TM. (**3**) Two tips retract to unload the preload force. (**4**) Rapidly retracting tips to release the TM.

The release procedure of the TM and dual-sided tips has undergone four main stages: (1) At the beginning of the experiment, the TM with a mass of 2.4 kg experiences the free oscillation both in simple pendulum motion and torsion balance motion. Then, two tips on opposite sides move slowly to the surface of gold-coated patches on the TM and make slight contact to grab the TM. (2) After about 10 s, a preload force of 0.3 N is applied to the TM to represent the in-orbit state of the TM constrained by tips, as shown in Figure 2B(c), and the TM quickly stabilizes. (3) The preload force is maintained for 30 s. Subsequently, two tips are slightly retracted to unload the preload force until the state of contact with the residual load to the order of $10^{-3}$ N is reached. (4) Finally, the tips are rapidly retracted to release the TM, and the displacement and angular displacement are measured to obtain the transferred momentum and angular momentum. The typical data of the simple pendulum motion and torsion balance motion of the TM mock-up, along with the signal of a preload force of 0.3 N applied to the TM, are shown in Figure 12a, Figure 12b, and Figure 12c, respectively.

It is evident that during the grab, load, and unload phases, the TM experiences slight vibrations attributed to the motion of the tips. However, the applied contact preload needs to be maintained for a certain period of time (at least 10 s), which should correspond to the actual state of the task. During the load phase, the TM position shifts are all less than 0.2 μm, which will result in a variation of the same length of the piezo extension, leading to a contact force uncertainty of 0.01 N, far less than 0.3 N. Due to the high-precision alignment of the two tips, the rotation of the TM, which is 0.01 mrad in the typical data, has a much smaller effect on the piezo extension than the effect of positional movement, so it can be ignored. During the unload phase, the TM is in a stable state and still maintains critical contact with the RT, with an uncertainty of less than 0.20 μm in position. Therefore, in the real-time monitoring of TM status, it can be ensured that the TM will not undergo premature detachments. After releasing, the amplitude of the simple pendulum motion caused by the transferred momentum is less than 1 μm, and the amplitude of torsion balance motion caused by the transferred angular momentum is less than 20 μrad. The typical parameters of the TM and the error analysis are shown in Table 2.

Using the measured periods of simple pendulum motion and torsion balance motion shown in Figure 11, the signal after release can be fitted, and the resulting curve is used as the motion measurement value. Taking the typical data in Figure 12 as an example, the sample point that starts sinusoidal oscillation and exceeds the ±3σ interval will be taken as the release sample, corresponding to the release time $t_{0}$. Then, the least squares method is used to perform sine function-fitting on the displacement and angle data after release, and the resulting curves are used as the motion displacement. Considering that the displacement signal also contains frequency components of torsional motion, the fitting functions are as follows:

(21) $$x\left(t\right)=a_{s}\sin\left(\omega_{x}t+b_{s}\right)+a_{t}\sin\left(\omega_{\theta }t+b_{t}\right)+c,$$

(22) $$\theta \left(t\right)=d_{t}\sin\left(\omega_{\theta }t+e_{t}\right)+h.$$

**Figure 13:**
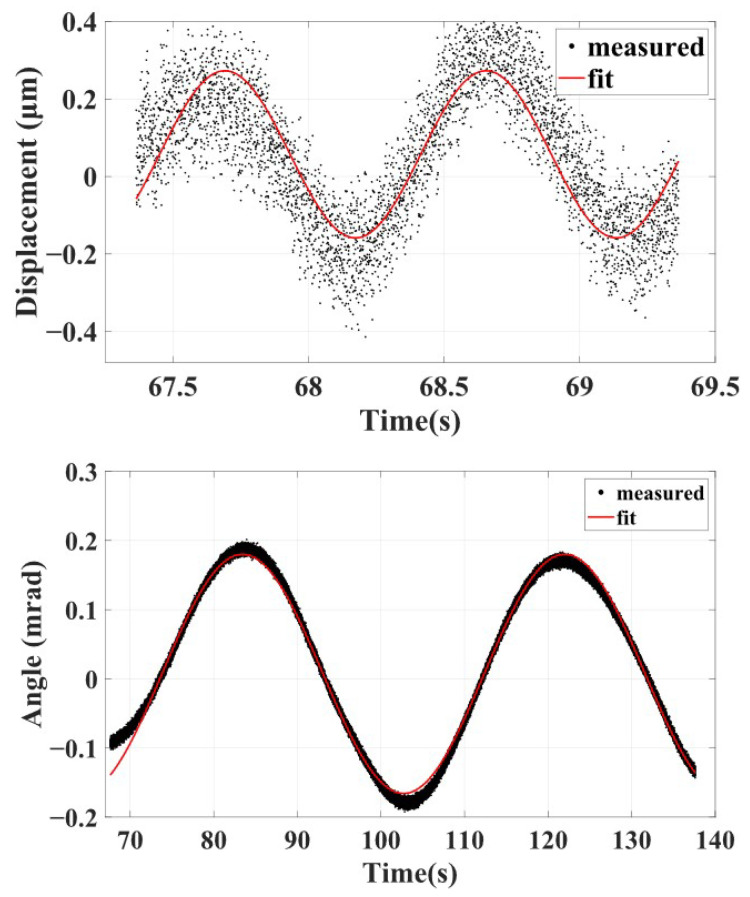
Data fitting curves of displacement and angle signals after the release process.

The fitting curve is shown in Figure 13, and the expressions for the displacement signal and angle signal of the pendulum motion are as follows:

(23) x(t)=0.215×sin(3.262t−1.582)μm,

(24) θ(t)=0.173×sin(0.163t+0.537)mrad.

**Figure 14:**
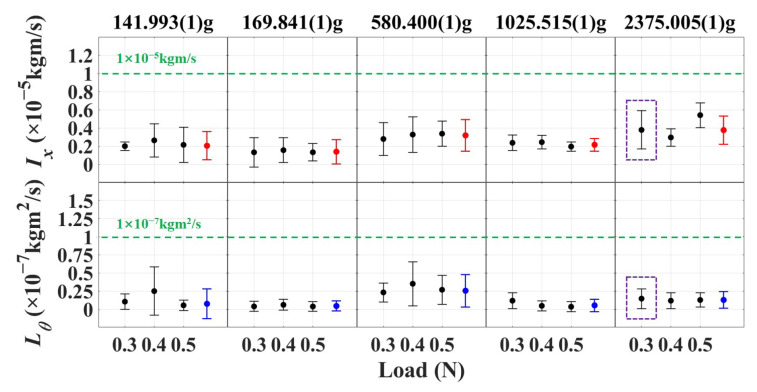
The experimental results with different kinds of TM mock-up under preloads of 0.3 N, 0.4 N, and 0.5 N. Each testing condition is repeated for 7 segments. The red dots and blue dots represent the average transferred momentum and angular momentum values of each type of TM mock-up, respectively. The green dashed lines are the release requirements. The purple dashed boxes are the experimental results of tungsten TM mock-up with a preload force of 0.3 N.

The maximum amplitudes and initial position are represented as $x_{m}=0.215\left(5\right)\;\mu$m, $x_{0}=x\left(t_{0}\right)=-0.058\left(4\right)\;\mu$m, $\theta_{m}=0.173\left(1\right)$ mrad, and $\theta_{0}=\theta \left(t_{0}\right)=-0.139\left(1\right)$ mrad. Taking into account the impact of bias on $x_{0}$ during the estimation of the release sample, an upper limit of 0.5 μm is selected for $\Delta x_{0}$, as shown in Table 2. According to Equations (17) and (19), the momentum and angular momentum for this typical release test are $0.16\left(9\right)\times 10^{-5}\;\mathrm{kgm}/s$ and $0.17\left(1\right)\times 10^{-7}\;{\mathrm{kgm}}^{2}/s$, respectively. The average of the experimental results of the seven segments are shown in the purple dashed boxes of Figure 14, showing that the released momentum is $0.38\left(21\right)\times 10^{-5}\;\mathrm{kgm}/s$ and the angular momentum is $0.15\left(14\right)\times 10^{-7}\;{\mathrm{kgm}}^{2}/s$.

Furthermore, given the potential variability of the contact load around the design value during the actual alternation process from the plunger to the tip, higher preload forces of 0.4 N and 0.5 N have been adopted to investigate the influence of different preload forces of dual-sided tips. This approach is intended to ensure the reliability of the design. Meanwhile, considering the different requirements of TM masses for different space missions, two kinds of hollow aluminum alloy TM with masses of 141.993(1) g and 169.841(1) g, a titanium alloy TM with a mass of 584.400(1) g, and a stainless steel TM with a mass of 1025.515(1) g have also been respectively employed to replace the tungsten TM to verify the dual-sided tips’ release scheme. When conducting error analysis, it is also found that the heavy mass TM has stricter error requirements for other parameters in the experiment. The experimental results obtained from using the same releasing-and-testing process as the tungsten TM mock-up are illustrated in Figure 14. The red dots represent the average transferred momentum values of each type of TM mock-up with different masses. Similarly, the blue dots represent the average transferred angular momentum of each TM mock-up. The experimental results demonstrated that there was no significant difference in the release results under the three preload force conditions, which indicates that the unloading process prior to release effectively mitigates the effect of the preload force. The variations observed in the release test results are likely attributed to inconsistencies in the mechanical behavior of the release system. One of the primary factors contributing to these variations is the potential lack of perfect synchronization in the retraction action of the release mechanism. If the retraction process is not fully synchronized between the two sides, the force profiles exerted by the release mechanism may exhibit slight asymmetries. These minor discrepancies can be amplified during the release process, leading to variations in the displacement or motion of the pendulum and, consequently, deviations in the measured results. During the release test of time intervals, there was a slight increase in the release results compared to the simultaneous release, which indicates that the asynchronous release of the release mechanisms on both sides is indeed one of the influencing factors. During our on-ground testing, the experimental results from different configurations are found to be consistent within the variation of the measurements, and all meet the release requirements of $1\times 10^{-5}\;\mathrm{kgm}/s$ for the momentum and $1\times 10^{-7}\;{\mathrm{kgm}}^{2}/s$ for the angular momentum represented as green dashed lines.

Ideally, the dual-sided RTs should simultaneously retract, denoted as $\Delta \tau_{0}$, with the time stability of data acquisition systems of 10 ns. However, considering the consistency and stability of the control system of the release mechanism, as well as complex spatial environmental disturbances, asynchronous retraction of the dual-sided RTs occur under normal conditions. When the time delay occurs during the release process, the residual contact load becomes directly converted into thrust acting on the TM, generating transfer momentum as described by Equation (20). With the expected worst-case residual load of approximately 10 mN, the permissible time delay must be strictly maintained below 1 ms to meet operational requirements. Consequently, release testing across the full temporal range of Δτ from 0 to 1 ms is essential to validate whether the TM’s post-release state remains within specified performance boundaries. By controlling the driving voltage, the time interval between the retractions of tips can be adjusted. In order to distinguish the release sequence at the same time interval, $\Delta \tau_{+}$ indicates that RT B retracts earlier, while $\Delta \tau_{-}$ indicates that RT A retracts earlier.

**Figure 15:**
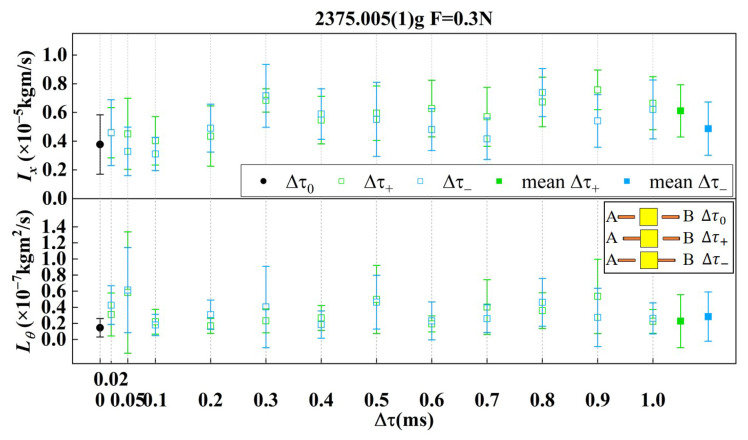
The testing results of the transferred momentum of the tungsten cubic TM mock-up through the time interval release process. $\Delta \tau_{0}$ represents a set of testing results from Figure 14. Twelve different Δτ values are selected for testing, ranging from 0.02 ms to 1 ms, with the total error of each group mainly coming from the error of the device. Each experimental condition is repeated for 6 segments. The measurement results for both $\Delta \tau_{+}$ and $\Delta \tau_{-}$ are in good agreement with each other. Within the above time lag range, the momentum measurement results are small and the error is relatively large; the impulse dependence on the time lag is not significant.

The testing results of the transferred momentum of the tungsten cubic TM mock-up with the preload force of 0.3 N of dual-sided tips through time interval release process are shown in Figure 15. The hollow square points indicate the value of each kind of time interval from 0 to 1 ms. The solid square points indicate the mean value of $\Delta \tau_{+}$ and $\Delta \tau_{-}$ of transferred momentum and angular momentum. Both of them are in good agreement with the value of the black circle dots $\Delta \tau_{0}$, which represent the simultaneous retraction of the two tips. In the experiment, the theoretical residual contact load is in the order of mN (<$10^{-2}$ N). Consequently, when a delay of less than 1 ms is introduced, the impact on momentum remains less than $1\times 10^{-5}\;\mathrm{kgm}/s$, which is consistent with the measurement results. And due to the relative measurement error, the value of momentum cannot be accurately distinguished within a 1 ms time interval, so the experimental measurement results do not show a significant correlation with Δτ. Furthermore, release tests with time intervals exceeding 1 ms were conducted at intervals of 2 ms, 5 ms, and 10 ms. The measured release momenta were approximately $1.71\left(43\right)\times 10^{-5}\;\mathrm{kgm}/s$, $3.16\left(69\right)\times 10^{-5}\;\mathrm{kgm}/s$, and $6.24\left(94\right)\times 10^{-5}\;\mathrm{kgm}/s$, respectively. These results demonstrate a clear linear relationship between the release momentum and the time delay. The findings further validate that a time delay requirement of less than 1 ms is reasonable and should be considered a critical design criterion for the release process.

## 5. Discussion

The gravitational reference sensor is one of the payloads in the TianQin space-borne gravitational wave detection project with a massive test mass and large gap between the TM and electrode frame. A dedicated three-level prototype structural locking-and-release mechanism has been designed to lock the TM during launch phase and release it before the scientific phase. The release mechanism driven by a set of piezoelectric stack actuators has been assembled and used in on-ground testing.

A pendulum system has been developed in order to investigate the release process. The mock-up of the TM is entirely consistent with the size and mass of the TianQin TM, and the dual-sided release tips apply preload force to constrain the TM mock-up. The unloading process prior to release reduces the residual contact load to the order of $10^{-3}$ N. Finally, the TM mock-up is released by quickly retracting the release tips. The transferred momentum and angular momentum are estimated from the free oscillations as $0.38\left(21\right)\times 10^{-5}\;\mathrm{kgm}/s$ and $0.15\left(14\right)\times 10^{-7}\;{\mathrm{kgm}}^{2}/s$, respectively. The experiments also utilize the cubic TM mock-ups with the same volume but varying masses ranging from 0.1 kg to 2.4 kg, ensuring representative release results. The release experiments verified the possible preloading parameter range of 0.3 N to 0.5 N, requiring a time interval of less than 1 ms. The experimental results under different conditions are listed in Table 3, which presents a series of on-ground tests, and the final results meet the release requirements, demonstrating the great performance of the dual-sided release mechanism.

The stability of the release mechanism is examined by varying the initial release conditions. The experimental results exhibit great repeatability, and are consistent with design expectations. The residual error is primarily attributed to statistical errors, as detailed in Table 2. These minor differences mainly come from the incomplete consistency during the retraction process of the release tip, where slight mechanical misalignments or asymmetries between release mechanisms introduce non-ideal force components. Such micro-scale imperfections become dynamically amplified during release, leading to observable variations in the pendulum’s displacement characteristics and consequent momentum measurements. During experimental testing, synchronized release is achieved by simultaneously delivering operational commands through the voltage control circuit to discharge the piezoelectric stack voltages, driving the tips to leave the TM, which is consistent with the in-orbit operation process. To quantitatively assess potential release asynchrony induced by external factors, controlled time delay is systematically introduced in the experimental configuration to evaluate their impact on release dynamics. Notably, the asynchronous retraction of the tips still complies with the release requirements, which indicates that the designed dual-sided release mechanism possesses a certain level of fault tolerance. On-ground testing of the dual-sided release mechanism provides confidence that the mechanism meets requirements and allows for a detailed prediction of the in-orbit TM release. Future work will involve further experimental tests to validate the influence of metal cold welding on the release process under vacuum conditions.

## Figures and Tables

**Table 1:** The TM release state requirements for each condition.

State	Value
Initial release position	≤±400μm
Linear velocity	≤±5μm/s
Transferred momentum	≤$10^{-5}\;\mathrm{kgm}/s$
Initial release angle	≤±2.5mrad
Angular velocity	≤±150μrad/s
Transferred angular momentum	≤$10^{-7}\;{\mathrm{kgm}}^{2}/s$

**Table 2:** Contributions of various experimental parameters to the main error budget. The parameters of “Release Tips” provide the uncertainty of the alignment between the release mechanisms on both sides of the TM. And the parameters of “Relative Position” provide the alignment of the release mechanism relative to the direction of pendulum motion. In addition, the “TM equilibrium position offset” means the displacement of the pendulum from the equilibrium center position at the initial moment of release.

	Parameter	Uncertainty	Momentum $\delta I_{x}$	Angular Momentum $\delta L_{\theta }$
Pendulum	Length of fiber	1 mm	$5.8\times 10^{-9}\;\mathrm{kgm}/s$	$2.8\times 10^{-11}\;{\mathrm{kgm}}^{2}/s$
	Mass of TM	10 mg	$4.2\times 10^{-11}\;\mathrm{kgm}/s$	$1.0\times 10^{-13}\;{\mathrm{kgm}}^{2}/s$
Release Tips	RT axis alignment	20 μm	$6.2\times 10^{-9}\;\mathrm{kgm}/s$	$4.0\times 10^{-10}\;{\mathrm{kgm}}^{2}/s$
	RT axis angle	20 mrad	$2.0\times 10^{-9}\;\mathrm{kgm}/s$	$5.0\times 10^{-9}\;{\mathrm{kgm}}^{2}/s$
Relative Position	Displacement of RT relative to TM	0.1 mm	$1.6\times 10^{-8}\;\mathrm{kgm}/s$	$1.0\times 10^{-9}\;{\mathrm{kgm}}^{2}/s$
	Angle of RT relative to TM	20 mrad	$2.0\times 10^{-9}\;\mathrm{kgm}/s$	$4.0\times 10^{-9}\;{\mathrm{kgm}}^{2}/s$
	TM equilibrium position offset	0.5 μm	$1.0\times 10^{-8}\;\mathrm{kgm}/s$	$1.3\times 10^{-10}\;{\mathrm{kgm}}^{2}/s$
System Noise	Displacement detector	-	$4.0\times 10^{-8}\;\mathrm{kgm}/s$	$7.0\times 10^{-10}\;{\mathrm{kgm}}^{2}/s$
	Ground vibration	-	$3.8\times 10^{-7}\;\mathrm{kgm}/s$	$3.1\times 10^{-9}\;{\mathrm{kgm}}^{2}/s$
Data Processing	Amplitude	-	$1.1\times 10^{-7}\;\mathrm{kgm}/s$	$3.6\times 10^{-10}\;{\mathrm{kgm}}^{2}/s$
	Phase	-	$9.0\times 10^{-9}\;\mathrm{kgm}/s$	$8.4\times 10^{-11}\;{\mathrm{kgm}}^{2}/s$
	Statistical error	-	$2.0\times 10^{-6}\;\mathrm{kgm}/s$	$1.2\times 10^{-8}\;{\mathrm{kgm}}^{2}/s$
Combined Uncertainty			$2.1\times 10^{-6}\;\mathrm{kgm}/s$	$1.4\times 10^{-8}\;{\mathrm{kgm}}^{2}/s$

**Table 3:** The experimental results under different conditions.

Mass of TM	Time Interval	Preload Force	Momentum (kgm/s)	Angular Momentum (${\mathrm{kgm}}^{2}/s$)
141.993(1) g	$\Delta \tau_{0}$	0.3–0.5 N	$0.20\left(16\right)\times 10^{-5}\;$	$0.07\left(21\right)\times 10^{-7}\;$
169.841(1) g	$\Delta \tau_{0}$	0.3–0.5 N	$0.14\left(13\right)\times 10^{-5}\;$	$0.04\left(7\right)\times 10^{-7}\;$
584.400(1) g	$\Delta \tau_{0}$	0.3–0.5 N	$0.32\left(17\right)\times 10^{-5}\;$	$0.26\left(22\right)\times 10^{-7}\;$
1025.515(1) g	$\Delta \tau_{0}$	0.3–0.5 N	$0.21\left(7\right)\times 10^{-5}\;$	$0.05\left(8\right)\times 10^{-7}\;$
2375.005(1) g	$\Delta \tau_{0}$	0.3–0.5 N	$0.38\left(15\right)\times 10^{-5}\;$	$0.13\left(12\right)\times 10^{-7}\;$
2375.005(1) g	$\Delta \tau_{+}$	0.3 N	$0.61\left(18\right)\times 10^{-5}\;$	$0.23\left(33\right)\times 10^{-7}\;$
2375.005(1) g	$\Delta \tau_{-}$	0.3 N	$0.49\left(18\right)\times 10^{-5}\;$	$0.28\left(31\right)\times 10^{-7}\;$

## Data Availability

The data that support the findings of this study are available from the corresponding author upon reasonable request.
